# A Greedy Algorithm-Based Stem Cell LncRNA Signature Identifies a Novel Subgroup of Lung Adenocarcinoma Patients With Poor Prognosis

**DOI:** 10.3389/fonc.2020.01203

**Published:** 2020-08-11

**Authors:** Seema Khadirnaikar, Annesha Chatterjee, Pranjal Kumar, Sudhanshu Shukla

**Affiliations:** ^1^Department of Biosciences and Bioengineering, Indian Institute of Technology Dharwad, Dharwad, India; ^2^Department of Electrical Engineering, Indian Institute of Technology Dharwad, Dharwad, India

**Keywords:** lung adenocarcinoma, lncRNA, embryonic stem cells, immune cells, greedy algorithm

## Abstract

Cancer stem cells play an essential role in therapy response and aggressiveness of various cancers, including lung adenocarcinoma (LUAD). Interestingly it also shares many features of embryonic stem cells (ESCs). Recently, long non-coding RNAs (lncRNAs) have emerged as a critical regulator of cell physiology. Here, we used expression data of ESCs, LUAD, and normal lung to identify 198 long non-coding hESC-associated lncRNAs (hESC-lncRNAs). Intriguingly, K-means clustering of hESC-associated lncRNAs identified a subgroup of LUAD patients [undifferentiated LUAD (uLUAD)] with high stem cell–like characteristic, decreased differentiation genes expression, and poor survival. We also observed that the uLUAD patients had overexpression of proteins associated with cell proliferation. Interestingly, uLUAD patients were highly enriched with the stemness-related gene sets, and had higher mutation load. A notable result observed was high infiltration of T cells and a higher level of neopeptides in uLUAD patients, making these patients an optimal candidate for immunotherapy. Further, feature selection using greedy algorithm identified 17-hESC-lncRNAs signature, which showed significant consistency with 198 hESC-lncRNAs–based classification, and identified a group of patients with high stem cell–like characteristic in the 10 most common cancer types and CCLE cell lines. These results suggest the conventional role of hESC-lncRNAs in stem cell biology. In summary, we identified a novel subgroup of LUAD patients (uLUAD) using a set of hESC-lncRNAs. The uLUAD patients had high stem cell–like characteristic and reduced survival rate and may be referred for immunotherapy. Furthermore, our analysis also showed the importance of lncRNAs in cancer and cancer stem cells.

## Introduction

Lung adenocarcinoma (LUAD) is a primary subtype of lung cancer with an abysmal survival rate (1–3). The majority of LUAD patients are diagnosed at a later stage and are medicated with radiation and chemotherapy irrespective of heterogeneous disease (4, 5).

Recently, advancement in immunotherapy has proved to be useful for the treatment of LUAD patients (6–8). However, not all patients respond to immunotherapy efficiently (8). Thus, it is imperative to identify the novel subgroups of LUAD patients for better treatment strategies.

Experimental results and bioinformatics analysis of existing high-throughput data have shown that cancer stem cells (CSCs) play a crucial role in the determination of aggressiveness, response to the drug, and resistance to various kinds of therapies in many cancer types, including LUAD (9, 10). It has been hypothesized that carcinogenesis and early development of embryo share molecular similarities, and dedifferentiation leads to the pluripotent nature of cancer cells (11). Additionally, many factors associated with reprogramming in the embryonic state are implicated in cancers (11, 12). These observations also suggest that carcinogenesis and pluripotency share activation of common signaling pathways (11, 12).

Recently, long non-coding RNAs (lncRNAs) have been implicated in various aspects of cancer development (13, 14). Previous findings have shown that lncRNAs play a significant role in regulating pluripotency in ESCs (15–17). However, a comprehensive analysis to identify the lncRNAs that regulate pluripotency and carcinogenesis must be explored.

In the current study, we have utilized the ESC RNA Sequencing (RNA-Seq) data and The Cancer Genome Atlas (TCGA) cancer patients' data to identify and catalog the lncRNAs with a potential role in cancer development and progression. Further, we applied a greedy algorithm to propose a signature for the identification of a subclass of LUAD patients with high stem cell–like characteristics and poor survival. We also validated the utility of this signature in other solid tumor types. Lastly, we concluded that cancer cell lines with high stem cell–like characteristics, as identified by the lncRNA signature, showed high resistance to various kinds of chemotherapy, suggesting that patients with high stem cell–like characteristics may require an alternative approach for more effective therapy.

## Materials and Methods

### Patients, RNA-Sequencing Data, and Expression Analysis

Level 3 count and FPKM RNA-Seq data for normal and tumors were obtained from TCGA–Genomic Data Commons (GDC) website. Lung cancer Michigan RNA-Seq data from previous publication were used as a validation set for expression data analysis and test set for survival analysis (18). RNA Sequencing data corresponding to embryonic stem cells (ESCs) H7, HUES1, HUES8, and HUES9 were obtained from GEO series accession number GSE102311 (19). Another set of RNA-Seq data for H9 and SC12-03 was downloaded from GEO series accession number GSE107552 (20). Clinical data for all the survival analysis were obtained from TCGA-GDC. The TCGA-LUAD patients also included 13 patients with large cell neuroendocrine carcinoma (LCNEC).

The stemness data for the TCGA patients were downloaded from the National Cancer Institute GDC website and the neoantigen data from the previous publications (21, 22). The reverse-phase protein expression data were downloaded from the Cancer Proteome Atlas (https://bioinformatics.mdanderson.org/public-software/tcpa/). For the normal human bronchial epithelial cells, the raw data (fastq files) were downloaded from NCBI SRA (SRA: SRP157114) using the SRA toolkit. fastp with the default values was used for quality control and adapter trimming (23). The reference genome and annotation files for GRCh37 were downloaded from Ensembl. STAR (2.7.3) was used for alignment and to obtain the count values. Coordinate sorted bam files obtained from STAR were used with StringTie (2.1.1) to obtain the FPKM values.

### Expression Analysis

Raw counts were used for all the differential expression analysis. All the genes with an expression of more than five average counts across the sample group were considered as “expressed,” and genes with fewer than five average counts across the sample groups were considered “not expressed.” To identify the hESC-lncRNAs, lncRNAs expressed in LUAD and not expressed in normal were pulled out, and differential expression analysis was performed using a *t*-test. Long non-coding RNAs with > 5-fold higher expression in LUAD compared to normal with 5% FDR and more than five average counts in ESCs were considered as positive stemness-associated lncRNAs. Similarly, lncRNAs with <0.2-fold differential expression in LUAD compared to normal with 5% FDR were considered as negative stemness-associated lncRNAs.

### Pathway Analysis, Network Analysis, and Gene Set Enrichment Analysis

To get a broad understanding of the function of the protein-coding genes (PcGs), pathway and network analyses were done using the Metascape tool (http://metascape.org) (24). Metascape performs the comparative analysis of datasets across multiple experiments. Gene Ontology (GO) analysis was also performed using Metascape. Gene set enrichment analysis (GSEA) software from Broad Institute (25) was used for both preranked and default GSEA.

### Survival Analysis

Clinical data for TCGA patients were downloaded from the TCGA-GDC server and merged with expression data. For Michigan dataset (test set), patients' data were obtained from previous publications (18, 26). All Kaplan–Meier (KM) analysis was done using the log–rank test in GraphPad software version 8.2.1 (San Diego, CA, USA). For Cox regression analysis, the *survival* package was used in the R environment. Hazard ratio (HR) with a *p* <0.05 was considered significant. For stemness prognostic score (SPS) calculation, the following equation was used: **SPS** = ∑ (**β**_*i*_ × expression_*i*_)

where β is Cox regression coefficient, and *i* is gene.

### Statistical Analysis

The comparison of two groups was made using a two-sided *t*-test, and the resulting lncRNAs with *p* <0.05 were considered significant. Similarly, three or more groups' comparison was made using two-sided analysis of variance, and the resulting lncRNAs whose *p* <0.05 was considered significant.

### Greedy Analysis and Random Forest Model Building

To remove the redundancy, greedy signature algorithm was used. For verification of the 17-lncRNA model, the random forest method was used.

The details of the greedy analysis and random forest model building are given in the Supplementary Methods.

## Results

### Expression Pattern of lncRNAs and PcGs in the Normal Lung, LUAD, and ESCs

**Figure 1:**
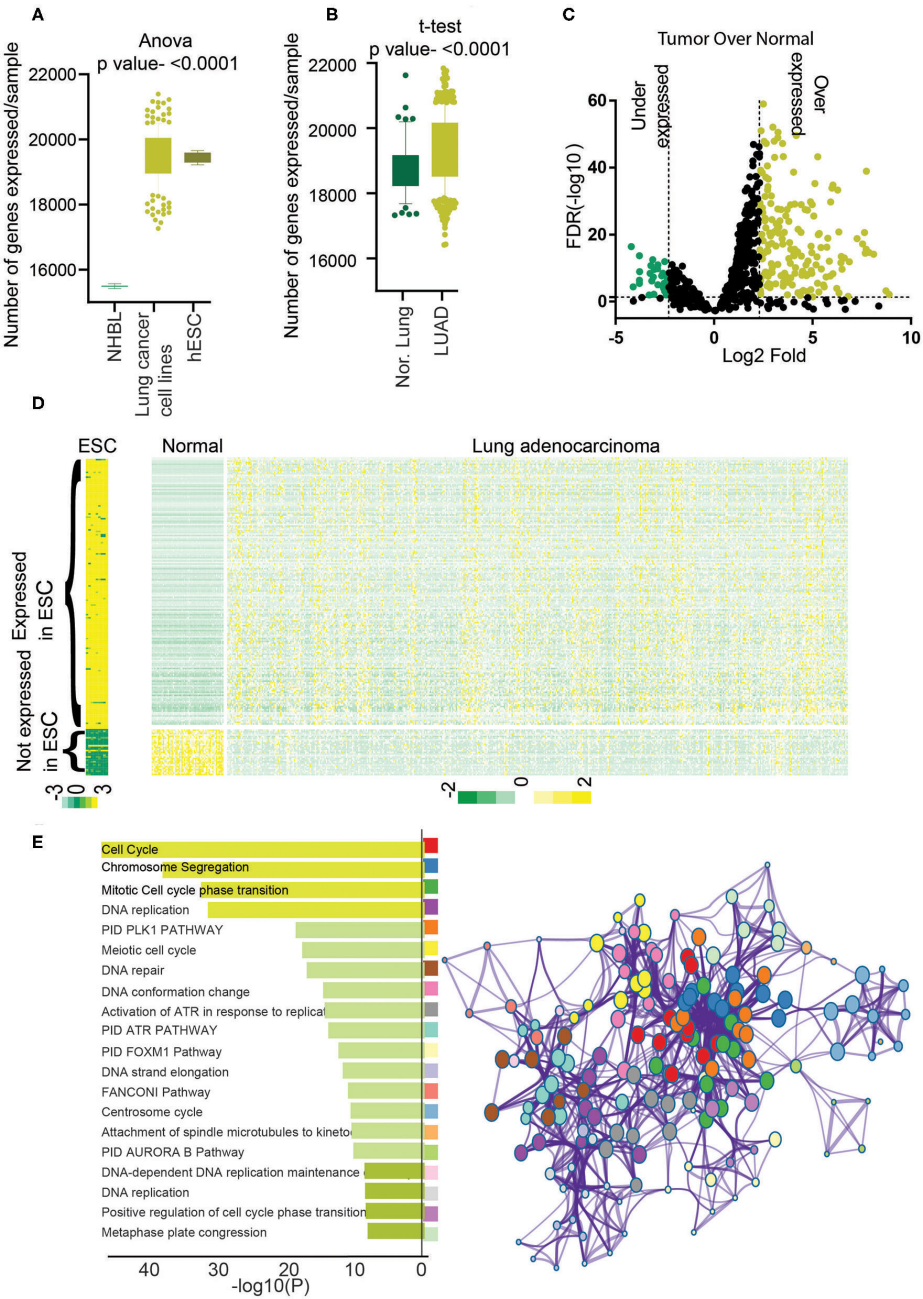
Identification of stemness-associated lncRNA. **(A)** The total numbers of expressed genes (>5 average count) in NHBL, lung cancer cell lines, and ESC were counted and plotted. An analysis of variance was done to find the significance. **(B)** The total number of expressed genes (>5 average counts) in normal lung and LUAD were counted and plotted. A *t*-test was performed to find the significance. **(C)** Differential expression analysis was performed, and lncRNAs with 5% FDR and more than 2-fold log2 difference were considered as differentially expressed lncRNAs. Volcano plot shows the overexpressed genes in yellow and underexpressed genes in green. **(D)** Heatmap showing the expression of differentially expressed 198 lncRNAs in ESCs, LUAD, and normal. Yellow shows high expression, and green shows low expression. **(E)** Gene Ontology analysis was performed using PcGs correlating with stemness lncRNAs (198), and enriched GO terms were plotted. The color of the bar indicates a higher significance. Metascape analysis was done using PcGs correlating with stemness lncRNAs (198) to identify the interactome network. Each dot represents one GO term, and the color key is given in the bar diagram.

Recent efforts on cataloging the transcripts expressed in human cells have shown that lncRNAs show a restricted expression pattern; that is, expression of lncRNAs shows higher tissue and lineage specificity than the PcGs (14). To understand the expression pattern of genes in normal human lung epithelial cells (NHLEs), normal lung (NL), LUAD, lung cancer cell lines (LCCs), and ESC expression data were analyzed as elaborated in the *Materials and Methods* section. Our analysis showed that significantly more genes are expressed in LCCs and ESCs compared to NHLEs (Figure 1A). Similarly, significantly more genes were expressed in LUAD compared to NL (Figure 1B). These observations suggest a comprehensive transcriptional dissimilarity among NHLEs, ESCs, and LCCs and between LUAD and NL.

### Identification of hESC-lncRNAs Associated lncRNAs in LUAD

To identify the lncRNAs associated with high stem cell–like characteristics in LUAD, first, we performed a differential expression analysis to identify the lncRNAs differentially expressed in LUAD compared to NL (*Materials and Methods*). The list of differentially expressed lncRNAs was then checked for their expression status in ESCs and lncRNA overexpressed in LUAD, and at least five average count in ESCs and downregulated in LUAD and fewer than five average count in hESCs were selected. This analysis identified a total of 198 lncRNAs associated with hESC and dysregulated in LUAD compared to NL, which we named as hESC-lncRNAs (Figure 1C, Supplementary Table 1). Among these lncRNAs, 169 lncRNAs were overexpressed, and 29 lncRNAs were underexpressed in LUAD to NL samples (Figures 1C,D). We checked the expression of 169 lncRNAs in normal human bronchial epithelium cells and found that average expressions of these lncRNAs were lower in NHEB than in ESCs (Supplementary Figure 1A). We also validated the expression of hESC-lncRNAs in another set of ESC (GSE107552) and LUAD samples (Michigan dataset) and found that 198 lncRNAs had similar expression patterns in another dataset as well (Supplementary Figures 1B,C). Further, to understand the functioning of hESC-lncRNAs, we performed pathway analysis using the PcGs, which had a high correlation (Pearson ρ > 0.3, *p* <0.05) with the selected 198 lncRNAs. The analysis identified that the PcGs with high correlation with hESC-lncRNAs were involved in the regulation of cell cycle, cell proliferation, DNA replication, DNA repair, and so on (Figure 1E). We also found that these pathways form a strong network in the cellular signaling (Figure 1E), suggesting a common role of hESC-lncRNAs in cellular proliferation and stem cell maintenance. These results also suggest that lncRNAs associated with high stem cell–like characteristics regulate various pathways involved in cell proliferation and growth. We also performed the canonical pathway analysis to identify the specific pathways regulating the cell cycle and proliferation. This analysis identified PLK1, Aurora, ATR, FOXM1, ATM, telomerase, ILK, P53, RB1, and MYC pathway associated with the genes correlating with 198 lncRNAs (Supplementary Figure 1D). Further, to understand the specific pathways regulated by individual lncRNA, we identified RP11-89K21.1 as one of the most LUAD-specific and prognostic lncRNAs among all the 198 hESC-lncRNAs (Supplementary Figures 2A,B). We then identified the PcGs with the most similar expression correlation to RP11-89K21.1 and performed Metascape analysis. Interestingly, we found that genes correlating with RP11-89K21.1 are associated with stem cell proliferation and Wnt signaling pathway regulation hESC-lncRNAs (Supplementary Figure 2C).

### High Stem Cell–Like Characteristic–Related lncRNAs Are Associated With the Prognostic Subtype of LUAD

**Figure 2:**
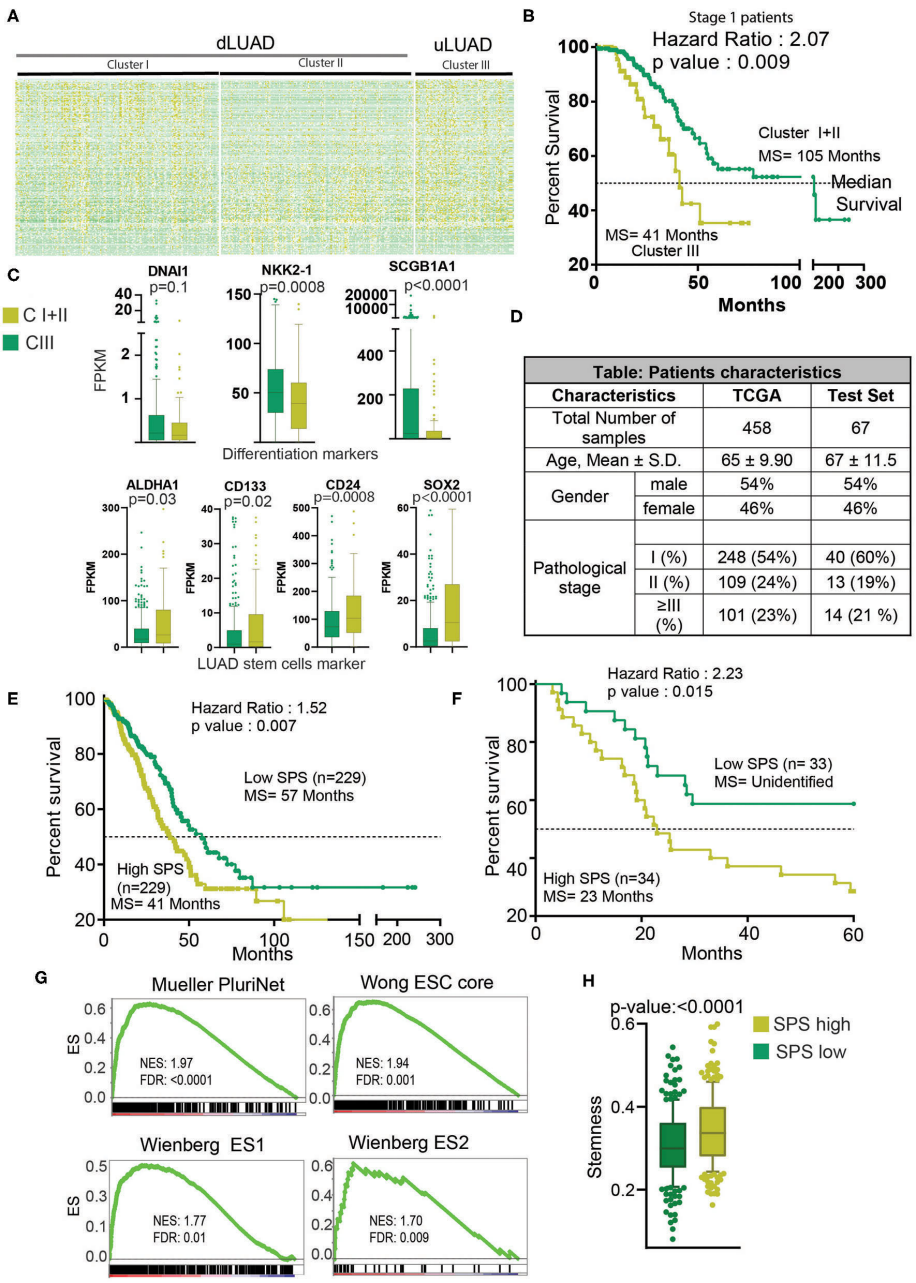
Clustering analysis identifies a novel cluster of uLUAD patients with the poor prognosis. **(A)** Heatmap of 198 lncRNAs in the clusters as identified by the K-means clustering algorithm. Yellow shows high expression, and green shows low expression. **(B)** A Kaplan–Meier plot showing the difference in survival between stage I patients of cluster I + II and cluster III as identified by K-means clustering. The *p*-value and hazard ratio were obtained by log–rank analysis. **(C)** Boxplots showing the expression difference in differentiation and stem cell markers. A non-parametric *t*-test was done to obtain the *p*-values. Bars show the standard deviation. **(D)** Table showing the patients' characteristics used in Cox regression analysis to identify the stemness-associated prognostic signature. A non-parametric test was done to show that patients from TCGA and test set did not have a significant difference in age. A Fisher exact test was done to show that the proportion of male and female and pathological stage distribution was similar in TCGA and test set. **(E)** Kaplan–Meier plot to show the significant difference in high- and low-SPS samples in TCGA patients set. Patients were divided into high and low SPS at the median. A log–rank test was performed to obtain the *p*-value and hazard ratio. **(F)** Kaplan–Meier plot to show the significant difference in high- and low-SPS patients in test set patients. Patients were divided into high and low SPS at the median. A log–rank test was performed to obtain the *p*-value and hazard ratio. **(G)** Gene set enrichment analysis showing enrichment of stemness gene sets in high- vs. low-SPS groups. **(H)** A boxplot showing the stemness scores of low-SPS and high-SPS patients, as described by Malta et al. (27). Bars show the standard deviation.

To further understand the interrelation of high stem cell–like characteristics–associated lncRNAs and LUAD subtype, we performed K-means clustering, which identified three clusters of patients, namely, clusters I, II, and III (Figure 2A, Supplementary Table 2). To delineate the clinical difference in these three clusters, we performed KM analysis. The KM analysis showed that patients belonging to cluster III had a significant poor survival compared to the other two clusters, clusters I and II (*p* = 0.026) (Supplementary Figure 3A). Thus, we combined clusters I and II patients for further analysis. As evident, we found that cluster III patients exhibited poor survival compared to cluster I + II patients (*p* = 0.015, HR = 1.55) (Supplementary Figure 3B). More importantly, in most clinically relevant stage I patients, cluster III patients showed significantly much poor survival compared to cluster I + II patients (*p* = 0.009, HR =2.07) (Figure 2B). Interestingly, we found that genes DNAI1, NKX2-1, and SCGB1A1, associated with the differentiation of different types of lung cells, were downregulated in cluster III patients compared to cluster I + II patients (Figure 2C). Similarly, ALDH1A1, CD133, CD24, and SOX2 markers of LUAD stem cells were upregulated in cluster III patients compared to cluster I + II patients, suggesting poor differentiation of cluster III patients (Figure 2C). Further, a preranked GSEA using genes upregulated during the early and late stage of differentiation of J1 ESC showed that these genes are significantly negatively enriched in cluster III compared to cluster I + II (Supplementary Figure 3C). These results suggest that cluster III patients' tumors are less differentiated and more aggressive compared to cluster I + II patients' tumors. Thus, we renamed the clusters as differentiated LUAD (dLUAD, cluster I + II) and undifferentiated LUAD (uLUAD, Cluster III) for further characterization (Figure 2A). To identify the stemness base- prognostic signature for the LUAD patients, Cox regression analysis was performed on the TCGA LUAD patients using 198 lncRNAs (Figure 2D). The analysis identified two stemness-associated lncRNAs (SATB2-AS1 and ABCA9-AS1) whose expression correlated with the survival of LUAD patients. Further, we calculated an SPS for each patient by combining the regression coefficient and expression of both the lncRNAs (Supplementary Table 3). In a univariate Cox regression analysis, SPS significantly correlated with survival (HR = 2.23, *p* = 6.99 ×10^−5^). Interestingly, in a multivariate analysis with tumor stage, SPS was an independent predictor of prognosis in TCGA-LUAD patients (*p* = 5.54 ×10^−5^) (Supplementary Figure 3D). Furthermore, to validate the prognostic utility of SPS, we utilized another set of 67 LUAD patients as the testing set (Figure 2D, Supplementary Table 4). Interestingly, SPS was found to be an independent prognosticator of survival in the testing set as well (*p* = 7.45 ×10^−3^) (Supplementary Figure 3E). More importantly, KM analysis showed a significant difference in survival of the patients with high and low SPS in both TCGA-LUAD (HR = 1.53, *p* = 5.00 ×10^−3^) and testing patient set (HR = 2.23, *p* = 1.50 ×10^−2^) (Figures 2E,F). Gene sets associated with stemness were significantly enriched in patients with high SPS and poor survival (Figure 2G). Stemness score, as identified by Malta et al. (27), was also significantly high for the high-SPS patients (*p* <0.0001) (Figure 2H).

### Characterization of Novel High Stem Cell–Like Characteristic–Associated lncRNA–Based Subtype of LUAD

**Figure 3:**
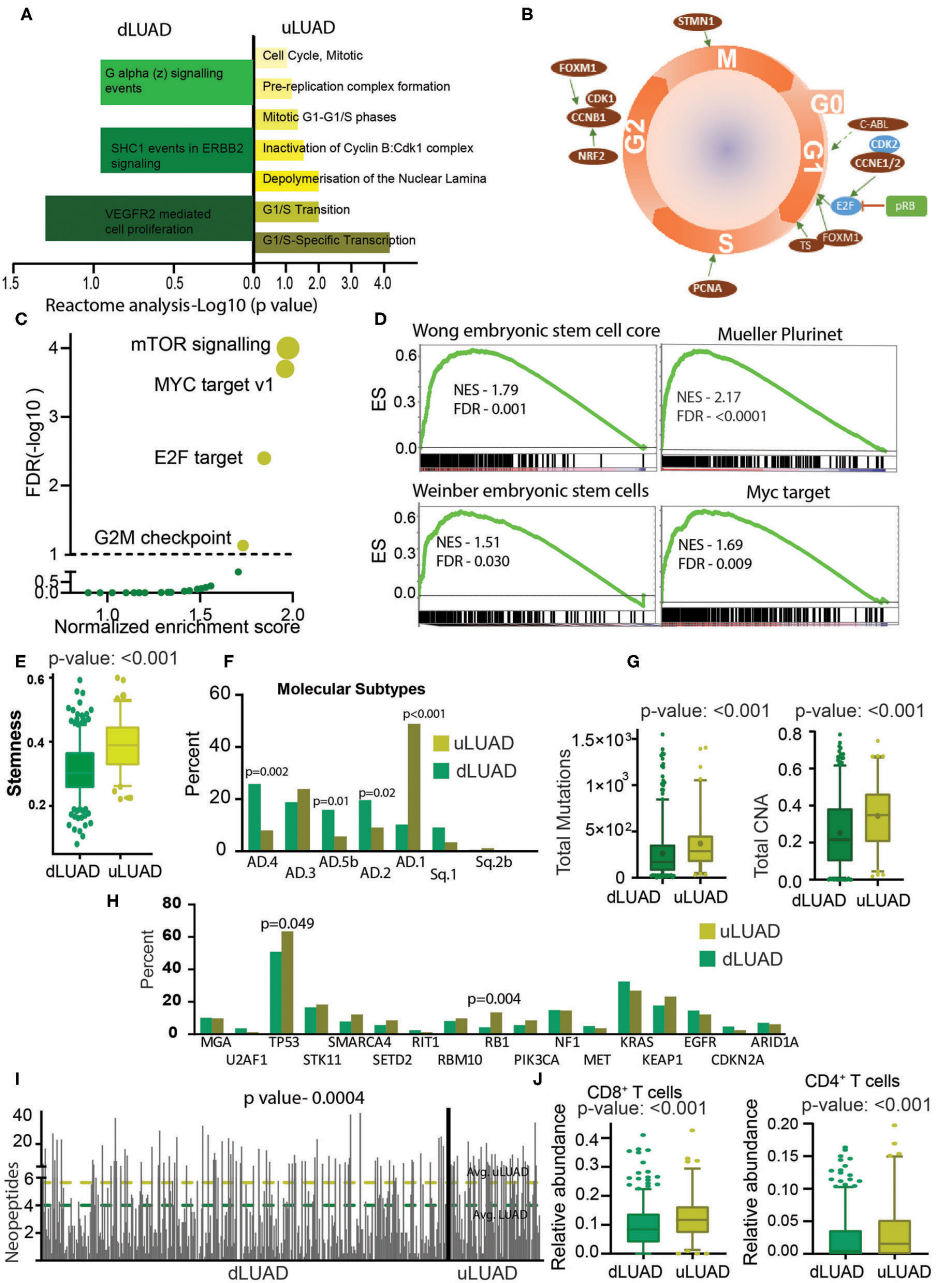
Undifferentiated LUAD patient subgroup is enriched in stemness and mutations. **(A)** A reactome analysis was done using the proteins differentially expressed between uLUAD and dLUAD patients. The bar plot was plotted using the significant reactome pathways. Pathways enriched in dLUAD are shown in green, and pathways enriched in uLUAD are shown in yellow. The color intensity indicates the *p*-value. **(B)** A model of the cell cycle was plotted, and proteins differentially expressed in uLUAD, and dLUAD were overlaid at the cell cycle stage where they function. The proteins indicated in red are overexpressed in uLUAD. The proteins shown in blue are significantly overexpressed at the RNA level, and proteins in green are significantly more mutated in uLUAD samples. **(C)** A GSEA was done using the hallmark gene set in dLUAD and uLUAD samples. The gene sets with <5% FDR were considered enriched. The significantly enriched gene sets are shown in yellow, and insignificant gene sets are shown in green. The size of the yellow bubble shows the enrichment score. **(D)** Gene set enrichment analysis was done using various gene sets obtained from stem cell markers. Normalized enrichment score and FDR are shown with GSEA plots. **(E)** A boxplot showing the stemness scores of dLUAD and uLUAD patients, as described by Malta et al. (27). Bars show the standard deviation. **(F)** Bar plot showing the enrichment of molecular subtypes of LUAD as identified by Chen et al. (28). **(G)** Boxplots showing the total mutation (left) and copy number aberrations (right) in dLUAD and uLUAD samples. The *p*-value was obtained using a *t*-test. Bars show the standard deviation. **(H)** Bar diagram showing the mutation pattern of commonly mutated genes in uLUAD and dLUAD patients. **(I)** A bar diagram showing the total neopeptide (neoantigen) in uLUAD and dLUAD samples. The green line shows the average neopeptides in dLUAD, and the yellow line shows the average neopeptide in uLUAD samples. The *p*-value was obtained from a *t*-test. Bars show the standard deviation. **(J)** A CIBERSORT analysis was done in dLUAD and uLUAD samples using absolute quantification settings. The *p*-value was obtained from a *t*-test. Bars show the standard deviation.

The RPPA data from TCGA were downloaded and analyzed to identify the direct changes in the proteins in dLUAD and uLUAD. This analysis identified 23 overexpressed and 18 underexpressed proteins (Supplementary Figure 4A, Supplementary Table 5). Reactome analysis was performed using the default setting for background correction to identify the function of these proteins. Interestingly, proteins overactive in uLUAD patients were associated with the cell cycle (Figure 3A). The analysis revealed that the majority of the proteins associated with uLUAD patients (c-ABL, CCNE1/2, FOXM1, TS, PCNA, NRF2, CCNB1, CDK1, FOXM1, and STMN1) were regulating cell cycle positively at all the cell cycle stages (Figure 3B, Supplementary Figure 4B). In contrast, the negative regulator of the cell cycle (CDK2, pRB, E2F) were downregulated (Figure 3B). Also, proteins differentially expressed in uLUAD compared to dLUAD appeared to have a close interaction in string analysis (Supplementary Figure 4C). Further global cancer analysis showed that most of the proteins overexpressed in uLUAD were highly active in various cancer types (Supplementary Figure 5A) compared to proteins underexpressed in uLUAD patients (Supplementary Figure 5B). These results suggest a high proliferative activity in uLUAD tumors.

To further illustrate the molecular differences between these novel subtypes of LUAD patients, we performed GSEA using the hallmark gene sets (Supplementary Table 6). Interestingly, we found that patients belonging to uLUAD showed enrichment of gene sets associated with oncogenic signaling (Figure 3C). More importantly, one of the significantly enriched gene sets was MYC targets. As MYC-mediated transcriptional changes are associated with pluripotency (29), we performed GSEA using stem cell marker gene sets and found that uLUAD samples were significantly enriched with genes related to the stem cells (Figure 3D). Furthermore, using the stemness score identified by Malta et al. (27), we showed that uLUAD patients have significantly higher average stemness score compared to dLUAD patients (Figure 3E) (27). Additionally, Metascape analysis using overexpressed genes in uLUAD compared to dLUAD identified the enrichment of pathways associated with cell cycle, DNA replication, and DNA damage response (Supplementary Figure 5C). In contrast, genes overexpressed in dLUAD were associated with cell adhesion and immune-related pathways (Supplementary Figure 5D). Recently, Chen et al. (28) have identified the nine molecular subtypes of NSCLC patients using a cluster of cluster analysis. We checked the enrichment of these molecular subtypes in dLUAD and uLUAD patients. Interestingly, dLUAD patients showed significantly higher enrichment of AD.4, AD.5b, and AD.2, whereas uLUAD patients showed the highest enrichment of AD.1 subtype, which shows poor differentiation of uLUAD tumors (Figure 3F). Notably, there was no significant difference in the enrichment of dLUAD and uLUAD patients in AJCC T, N, and M subtypes and LUAD stages (Supplementary Figures 6A–D). We also showed that there was no significant difference in the enrichment of dLUAD and uLUAD samples in histopathological subtypes of LUAD (Supplementary Figure 6E).

Recently, Tomasetti et al. (30) have suggested that tissues with high replication rates generate more random mutations, and these mutations are the most common cause of cancer. To validate this hypothesis, we compared the total somatic mutations in dLUAD and uLUAD. As expected, the mutation burden and copy number aberration are significantly higher in uLUAD samples (Figure 3G). Further analysis of specific mutations showed that Tp53 and RB1 were significantly more mutated in uLUAD compared to dLUAD (Fisher exact test *p* = 0.049 and 0.0004 for TP53 and RB1, respectively) (Figure 3H). More importantly, uLUAD patients showed a significantly higher number of neoepitopes compared to dLUAD patients, making these patients a better candidate for immunotherapy (Figure 3I). The immune response of cancer cells depends on the presence of neoepitopes and enrichment of CD8^+^ T cells, CD4^+^ T cells, and antigen-presenting cells (APCs) such as dendritic cells (31). CIBERSORT analysis was performed for both dLUAD and uLUAD samples to compare the enrichment of various immune cells in tumor milieu (Supplementary Table 7). Interestingly, uLUAD samples showed significantly higher enrichment of CD8^+^ and CD4^+^ T cells in uLUAD samples compared to dLUAD samples (Figure 3J) (31, 32). However, many other types of cells, including antigen-presenting dendritic cells, mast cells, M2 macrophages, and monocytes, were significantly enriched in dLUAD samples (Supplementary Figure 6F). The absence of APCs may be the reason for the weak immune activity of uLUAD samples despite the high neoepitopes load and presence of T cells.

### Identification of lncRNAs for High Stem Cell–Like Characteristics Signature in LUAD Patients Using the Greedy Algorithm

**Figure 4:**
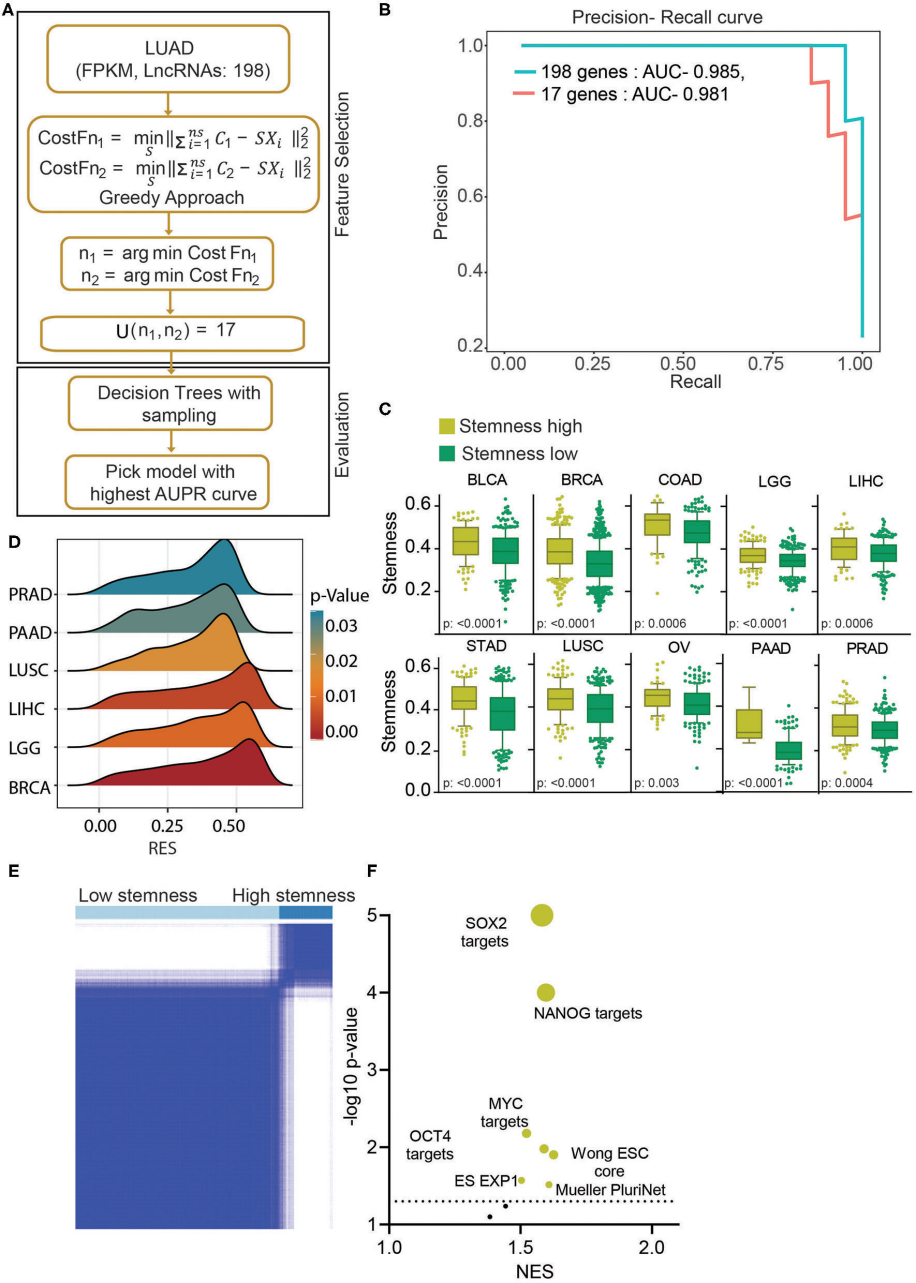
Stemness-associated lncRNA signature identifies high stemness patients and cell lines from various cell types. **(A)** A greedy algorithm was used to identify the stemness-associated lncRNA signature. This flowchart depicts the various steps involved in the analysis. **(B)** A precision–recall curve to show the consistent performance of 17 lncRNAs in classifying the stemness in LUAD patients compared to 198 lncRNA model. **(C)** Boxplots showing the difference in stemness in the top 10 cancers. The clusters were identified using K-means clustering of given cancers using 17 lncRNAs. The *p*-value was obtained using a *t*-test. Bars show the standard deviation. **(D)** A GSEA was done using the Muller PluriNet gene set in high and low stemness cluster of given cancer types. A ridge plot showing the running enrichment score and *p*-value in top tumor types. Red indicates a higher significance, and blue indicates lower significance. **(E)** The K-means clustering identified two clusters of cell lines based on the 17-lncRNA expression. **(F)** A GSEA was done in using stemness-associated gene set in high and low stem cell lines. The significantly enriched gene sets are shown in yellow, and insignificant gene sets are shown in black. The size of the bubble shows the *p*-value.

In earlier results, we have identified a novel subgroup of LUAD patients (uLUAD) with highly aggressive disease, most likely due to the presence of a higher fraction of LUAD stem cells. This subgroup of patients was identified using 198 hESC-lncRNAs with high expression in ESCs and LUAD. We hypothesized that not all 198 lncRNAs might be required for the high stem cell–like characteristics determination of the patients, and there may be redundancy. Hence, to identify a strong and non-redundant lncRNA-based signature, we performed feature selection analysis. Exhaustive search using all the possible combinations of the features is not a feasible solution as it is computationally complex. Therefore, we used a greedy forward feature selection approach where the model is built successively by adding one feature in each iteration, and the chosen feature will be the optimal feature in the current iteration (Figure 4A).

This analysis identified 17 discriminant hESC-lncRNAs as the optimum number of features to classify the uLUAD and dLUAD patients (Supplementary Method). Further, to check the discriminative ability of the selected hESC-lncRNAs classification, a model was built using a random forest algorithm and smote sampling (Figure 4A). To avoid overfitting, 10-fold cross-validation was repeated 10 times, and optimal hyperparameters were chosen by grid search. The precision–recall area under the curve of this model showed that 17 hESC-lncRNAs could classify the dLUAD and uLUAD patients without any significant degradation in performance as compared to 198 hESC-lncRNAs (Figure 4B). To further verify the significance of these 17 hESC-lncRNAs in high stem cell–like characteristics classification, we carried out K-means consensus clustering in the 10 most common tumor types using the optimum number of clusters as two. The clustering analysis showed that the 17-lncRNAs could classify various cancer types into high and low stem cell–like characteristics categories with a significant difference in stemness score as identified by Malta et al. (27) (Figure 4C). Moreover, a GSEA using Mueller PluriNet gene set (includes common characteristics of pluripotent cells from the different origin) found significant enrichment in six cancer types and enrichment approaching significance in the other four cancer types (Figure 4D, Supplementary Figure 7A). Differential expression analysis of matched normal vs. tumor showed a high expression of all the 17 signature lncRNAs in cancer (Supplementary Figure 7B). Receiver operating characteristic (ROC) analysis also showed that expression of all the 17-lncRNA could discriminate between matched normal and tumor with high specificity and sensitivity. Furthermore, ROC analysis of tumor vs. normal in general showed that these 17-lncRNAs could discriminate between tumor and normal with high specificity and sensitivity (Supplementary Figures 7B,C). Next, we performed K-means consensus clustering analysis using the selected 17 lncRNAs in CCLE cells to classify the cell lines based on their stemness. This analysis classified the cell lines into two clusters, namely, high stemness cell lines and low stemness cells lines (Figure 4E, Supplementary Figure 7B) The expressions of 17 classifying hESC-lncRNAs were significantly higher in high stem cell–like cell lines compared to low stem cell–like cell lines (Supplementary Figure 7D, Supplementary Table 8). As expected, gene sets associated with stemness were significantly enriched in cell lines classified as high stem cell–like compared to low stem cell–like cell lines (Figure 4F).

## Discussion

Recent experimental and clinical observations have shown that aggressiveness and drug resistance of cancers, including lung cancer, are sustained by CSCs (11, 33). Cancer stem cells share many characteristics with ESCs, which give rise to various properties including anchorage-independent growth, proliferation, metabolic requirements, inhibition of differentiation, and so on (11, 34, 35). Also, multiple studies have shown that the dedifferentiation of normal cells is one of the initial steps in carcinogenesis, and cancer cells have a similar molecular regulatory network as ESCs (35, 36). Similarly, lung cancer cells also show a higher expression of ESC-associated genes (12, 37–39). Validating this observation, we proved that LUADs share a much higher transcriptomic (including lncRNAs expression) overlap with ESCs compared to NL cells (Figure 1A). We also identified a group of 198 lncRNAs (hESC-associated lncRNA, hESC-lncRNAs) with high differential expression in ESCs and LUAD compared to NL. The PcGs with similar expression patterns to 198 lncRNAs indicated the involvement of high stem cell–like characteristics–associated lncRNAs in cell proliferation and other cancer-associated roles. We identified various pathways that are associated with PcGs with similar expression pattern to 198 lncRNAs (Supplementary Figure 1D). PLK1 pathway is essential for the initiation and completion of mitosis and thus required for cell proliferation (40). Similarly, the Aurora kinase pathway is one of the crucial pathways for successful cell division and proliferation of the cells (41). Also, Aurora-A is required for the maintenance of the ESC self-renewal and undifferentiated state (42). Aurora kinase B is also needed for the maintenance of telomerase activity and stem cells (43). FOXM1 pathway is required for cell proliferation, self-renewal, and tumorigenesis (44). Similarly, RB1-E2F is necessary for cancer cell growth, migration, self-renewal differentiation, and so on (45). These results suggest the role of hESC-associated lncRNAs in cell proliferation and differentiation.

Unsupervised clustering has been used to identify the novel subgroups of many cancer types (46–48). Here, we utilized the K-means clustering algorithm with high stem cell–like characteristics–associated lncRNAs to identify a unique subset of LUAD patients. These patients showed poor survival and lower expression differentiation markers such as DNAI1, NKX2-1, and SCGB1A1, and higher expression of stem cell markers such as ALDH1A1 and CD133 (37, 49, 50). The expressions of DNAI1, NKX2-1, and SCGB1A1 genes are required for the differentiation of many cell types, including secretory (club) cells (49, 51, 52). This observation suggests that the novel LUAD subgroup named as uLUAD cells is less differentiated and has high ESC-like properties. The poor differentiation is associated with various cancer hallmarks such as proliferation, replicative immortality, angiogenesis, higher metastasis, and so on (53). These properties make poorly differentiated cancers more aggressive with poor outcomes, as found in uLUAD patients. A Cox regression analysis identified two stemness-associated lncRNAs with a high correlation with survival. We developed a stemness lncRNA prognostic score (SPS) and proved the prognostic ability of SPS in two independent cohorts of samples (Figures 2F,G, Supplementary Figures 2C,D).

Many transcription factors, including MYC, play a vital role in stem cell biology (54, 55). MYC has been shown to induce ESC-like characters in normal and cancer cells (56, 57). We showed that the novel uLUAD patients had higher MYC activity. SOX4, another stem cell pluripotency factor, is also significantly more active in uLUAD samples compared to dLUAD samples. Furthermore, the direct ESC-related genes were also significantly enriched in uLUAD compared to dLUAD. These observations validated the high stem cell–like characteristics of uLUAD patients identified by stemness-associated lncRNAs. The high stem cell–like characteristics are linked with high cellular proliferation, which in turn causes more genetic instability and high mutation rate and copy number aberrations. We also found the activation of proteins involved in cellular proliferation (Figures 3A,B). This observation was further supported by the high mutational load of uLUAD patients (Figure 3F). The uLUAD patients also showed high neoantigens compared to dLUAD patients. Recently, Chen et al. (28) have classified NSCLC in nine genomic subtypes, that is, SQ.1, SQ.2a, SQ.2b, AD.1, AD.2, AD.3, AD.4 AD.5a, and AD.5b. We found that all the LUAD patients used in this analysis were enriched in five of nine subtypes, SQ.1, SQ.2b, AD.1, AD.2, AD.3, AD.4, and AD.5b. Undifferentiated LUAD patients' proportion was significantly higher in the AD.1 subtype. AD.1 subtype is associated with poor differentiation, association with LCNEC, and expression of CT antigens, confirming our findings. In comparison, dLUAD patients were considerably higher in AD.4, AD.5b, and AD.2 subtypes. AD.4 subtype is associated with high immune infiltration, lower neoantigen, and lower mutation rate. AD.5b subtype is associated with lower mutation rate and high mTOR pathway activation, whereas the AD.2 subtype is associated with the high immune cell and checkpoint pathway activation. All three subtypes (AD.4, AD.5b, and AD.2) are also associated with excellent survival. These results positively confirm our finding that dLUAD patients have lower neoantigen and show good survival compared to uLUAD patients. Other subtypes are associated with high SOX2 and CT antigen expression (SQ.1), high SOX2, CT antigen expression, better OS (SQ.2A), distinct methylation patterns compared to SQ.2a (SQ.2B), high immune cell infiltration, and CT antigen expression (AD.3). Recent reports have suggested that the presence of neoantigens is essential for the checkpoint inhibitor–mediated immune response of T cells (58). Interestingly, we also found that uLUAD patients have a higher level of CD8^+^ and CD4^+^ T cells in the tumor milieu. These observations suggest that uLUAD patients could be a significant group of patients for immunotherapy. Interestingly, checkpoint inhibitors such as CD274 are also overexpressed in uLUAD patients, making these tumors more immunoactive. However, we believe that the absence of APCs such as dendritic cells from uLUAD cells may be a responsible weak T-cell activity (Supplementary Figure 2E). Also, we did not find any association of stage and hESC-like characteristic, suggesting the expression of hESC-like lncRNAs is probably an early event in LUAD development.

To build a classification model that efficiently distinguishes uLUAD from dLUAD samples, there was a need to eliminate the redundant features and retain only the discriminant lncRNAs as building a model with redundant features not only increases the computational complexity but also may lead to overfitting. Using a greedy algorithm, we identified a list of 17 lncRNAs. As these lncRNAs had a very high expression in ESCs, we hypothesized that unsupervised clustering of other tumor types using these hESC-associated lncRNAs should identify subgroups of cancer with high stemness. Interestingly, the 17-hESC-associated-lncRNA signature identified the subgroups in the top 10 tumors with a significant difference in stemness. This observation suggested that these hESC-associated lncRNAs were involved in stemness determination in general. Further, the hESC-associated lncRNA signature also identified a group of cell lines with high stemness characters. These cell lines could prove to be a useful tool for stem cell research and drug discovery.

Here, we performed various *in silico* analyses to show the importance of lncRNA in stemness determination and prognosis. However, experimental validation of stemness-associated lncRNAs is essential to show the direct effect on stemness determination, and it is an important shortcoming of this study. Taken together, we have utilized a large set of tumor patients to identify the stemness-associated lncRNAs. We have also identified a subgroup of LUAD patients who showed a significant difference in survival and stem cell–like characteristics. We propose that the aggressiveness of these patients is due to the presence of CSCs. We also showed that these patients could be an important target for immunotherapy.

## Data Availability Statement

Publicly available datasets were analyzed in this study. This data can be found in The Cancer Genome Atlas (https://portal.gdc.cancer.gov/) and the NCBI Gene Expression Omnibus (GSE102311).

## Author Contributions

SK and SS designed the research. SK, AC, PK, and SS performed the experiments. SK, AC, and SS analyzed the data and wrote the manuscript. All authors contributed to the article and approved the submitted version.

## Conflict of Interest

The authors declare that the research was conducted in the absence of any commercial or financial relationships that could be construed as a potential conflict of interest.
