# Supplementary material for: A Greedy Algorithm-Based Stem Cell LncRNA Signature Identifies a Novel Subgroup of Lung Adenocarcinoma Patients With Poor Prognosis
Source: Front Oncol. 2020 Aug 11;10:1203. doi: 10.3389/fonc.2020.01203 (PMC7431877; doi:10.3389/fonc.2020.01203)

Supplementary Methods

Greedy analysis for feature selection

Building a model with redundant features not only increases the computational complexity but may also lead to over-fitting. Hence the need to eliminate redundant features and retain only the discriminant ones. Given the input features $\{x_1, x_2, x_3, \ldots, x_{198}\}$ the aim of feature selection is to pick a subset of features $\{x_1, x_2, x_3, \ldots, x_M\}$, where $M < 198$ by ensuring that the classification task is carried out efficiently.

We chose to carry out this task by greedy feed forward approach. The feed forward approach finds the solution incrementally and the greedy approach picks the locally optimal feature in each stage which is added to the existing model in each iteration. The optimal feature is the one which has the least value of the cost function and the addition of features is carried out till further addition does not strengthen the classification model (will not lead to any improvement in the performance of classification model). Here, the cost function is used to find the set-of-features that are at a minimum distance from centroids.

We formulated the cost function *(J)* as a convex optimization problem with the aim to find a sparse diagonal matrix *(S)* which minimizes the distance between the cluster centre *(C)* and the Fragments Per Kilobase of transcript per Million mapped reads (FPKM) values *(X)* for different samples. It can be represented as,

$$J(S,\lambda) = \min_{S} \left\| \sum_{i=1}^{ns} C_j - SX_i \right\|_2^2, \; j \in [1,2] \qquad (1)$$

where j represents cluster index, λ is the number of genes of interest, nr is the number of rows (LncRNAs) in sparse matrix (S), ns is the number of samples, and $X\ and\ C$ are the FPKM values and the centroids respectively for a given cluster.

Pseudocode for the greedy algorithm is given below:

Algorithm 1:

procedure Greedy Algorithm(λ, X, nr, ns)

$S \leftarrow 0$

$for\ z \leftarrow 1\ to\ \lambda\ do$:

$cost_{fn}[\quad] \leftarrow 0$

$for\ i \leftarrow 1\ to\ nr\ do$:

$S[i,i] \leftarrow 1$

temp $\leftarrow 0$

$for\ k \leftarrow 1\ to\ ns\ do$:

$temp \leftarrow temp + (C - SX[,k])$

$end\ for$

$cost_{fn}[i] \leftarrow temp$

$S[i,i] \leftarrow 0$

$end\ for$

$m = \left(arg\ min\left(cost_{fn}\right)\right)$

$S[m,m] \leftarrow 1$

$end\ for$

$return\ cost_{fn}$

end

Genes of interest were chosen based on the value of λ for each cluster which returned the least cost function. The increment in cost function indicates that any further addition of LncRNAs will not contribute any new information and hence will not help in classification.

As shown in **Supplementary Method Figure 1** the cost function was minimum at $\lambda = 10$ for

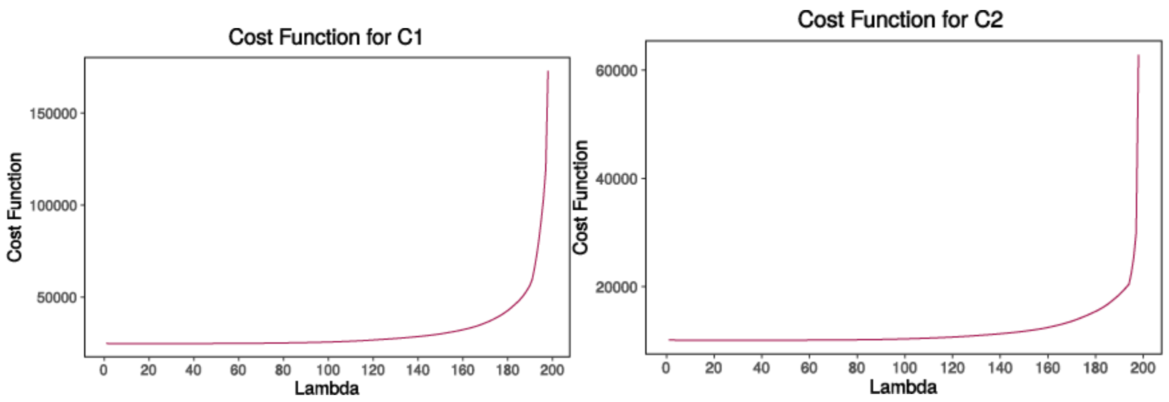

Supplementary Method figure 1

the first cluster and $\lambda = 15$ for the second cluster. Hence, we picked the top 10 genes and top 15 genes from the first and the second cluster respectively and the union of these returned 17 genes which we hypothesised to have the ability to classify the samples into high and low stemness groups.

To verify that the selected LncRNAs are able to carry out the classification efficiently, we built models based on decision trees (random forest and xgboost). Due to class imbalance, up, down and smote sampling was carried out and the resulting models were evaluated based on the area under the Precision-Recall (PR) curve. Grid search was used to pick the optimal hyper-parameters to tune the model. While building and evaluating the model the data was randomised and divided into training (70%) and testing (30%) sets. To avoid overfitting, we used 10 fold cross-validation and repeated it 10 times.

Models with all the combinations were built using 198 LncRNAs and the model that had the highest area under PR curve for testing set was further trained using the selected 17 LncRNAs.

The results are tabulated in **supplementary method Table 1 ---**. Area under the PR curve was 0.985 and 0.981 for model with 198 LncRNAs and 17 LncRNAs respectively. The decrement in areas is acceptable as there is always a trade-off between the accuracy and the size of the model.

Supplementary Method Table1							
**Sl. No.**	**AUC**	**Precision**	**Recall**	**F1 score**	**Model**	**Sampling**	**No. of Genes**
1	0.981	0.983	0.817	0.892	rf	down	198
2	0.971	0.959	1	0.979	rf	up	198
3	0.985	0.986	0.986	0.986	rf	smote	198
4	0.968	0.964	0.746	0.841	xgbTree	down	198
5	0.982	0.972	0.986	0.979	xgbTree	up	198
6	0.968	0.956	0.915	0.935	xgbTree	smote	198
7	0.981	0.97	0.915	0.942	rf	smote	17

Supplementary Figure

legends Supplementary

Figure 1:

A) The heatmap of 198 LncRNA showing the average expression of 198 LncRNAs in NHBE, ESC samples.

B) The heatmap of 198 LncRNA in another set of embryonic stem cells expression data (GSE107552). Yellow shows higher expression and green shows lower expression.

C) A volcano plot showing the differential expression of 198 LncRNAs in Michigan dataset with 6 normal and 67 tumor samples.

D) A bar plot showing the enriched pathways obtained from Metacore analysis using the 198 LncRNAs correlating PcGs as input.

Supplementary Figure 2

A) Box plot showing log transformed average count of RP1-89K21.1 in ESC, LUAD and normal.

B) Plot showing KM analysis in RP1-89K21.1 high and low group. Samples were divided at median. Log-rank test was performed to obtain p-value and hazard ratio.

C) GO analysis/Metascape was carried out with PcGs associated with RP1-89K21.1.

Supplementary Figure 3:

A) A Kaplan Meier analysis showing the poor survival of cluster III patients compared to cluster I and cluster II. The p-values were obtained using log-rank test.

B) A Kaplan Meier analysis showing the poor survival of cluster III patients compared to cluster I+II. The p-values were obtained using log-rank test.

C) GSEA analysis showing the enrichment of genes overexpressed during early and late

phase of embryonic development.

D) A forest plot showing the results of univariate and multivariate Cox regression analysis of shown factors in TCGA samples.

E) A forest plot is showing the results of univariate and multivariate Cox regression analysis of shown factors in test set samples.

Supplementary Figure 4:

A) Heatmap of differentially expressed protein from LUAD TCGA-RPA dataset. Yellow indicates higher expression and green is low expression.

B) Figure showing the Interaction map of gene and pathway of overexpressed genes uLUAD compared to dLUAD. The analysis was done using GSCAlite web application.

C) A network of protein differentially expressed in uLUAD compared to uLUAD from string database.

Supplementary Figure 5:

A) Global cancer activation of overexpressed proteins in uLUAD as identified in GSCA lite analysis.

B) Global cancer activation of under expressed proteins in uLUAD as identified in GSCA lite analysis.

C) Metascape analysis was done using upregulated genes in uLUAD compared to dLUAD samples and significantly enriched GO terms were converted to network layout and shown. The color code is given as key for Enriched GO terms.

D) Metascape analysis was done using downregulated genes in uLUAD compared to

dLUAD samples and significantly enriched GO terms were converted to network layout and shown. The color code is given as key for Enriched GO terms.

Supplementary Figure 6:

A) Bar plot showing the distribution of dLUAD and uLUAD samples in AJCC M, **B)** AJCC N and **C)** stage **D)** AJCC T stage and **E)** histopathological LUAD tumor types.

F) A heatmap of the immune cell absolute enrichment in dLUAD and uLUAD tumors. The FDR values are shown in brackets. The green represents lower enrichment, and yellow represents high enrichment.

Supplementary Figure 7:

A) A GSEA analysis was done using Mueller PluriNet gene set in high vs low stemness sample of given cancer types. The significantly enriched gene sets are shown in yellow and insignificant gene sets are shown in black. The size of the yellow bubble shows the p-value.

B) Heatmap showing the expression of LncRNAs in LUAD and matched normal. Table showing the t-test p-value and ROC analysis result of 17 LncRNA expression in LUAD and matched normal.

C) ROC analysis showing the ability of 17 LncRNAs to discriminate between tumor and normal with high specificity and sensitivity.

D) A Heatmap of seventeen LncRNAs in high and low cancer stem cell lines.

Supplementary Figure 1

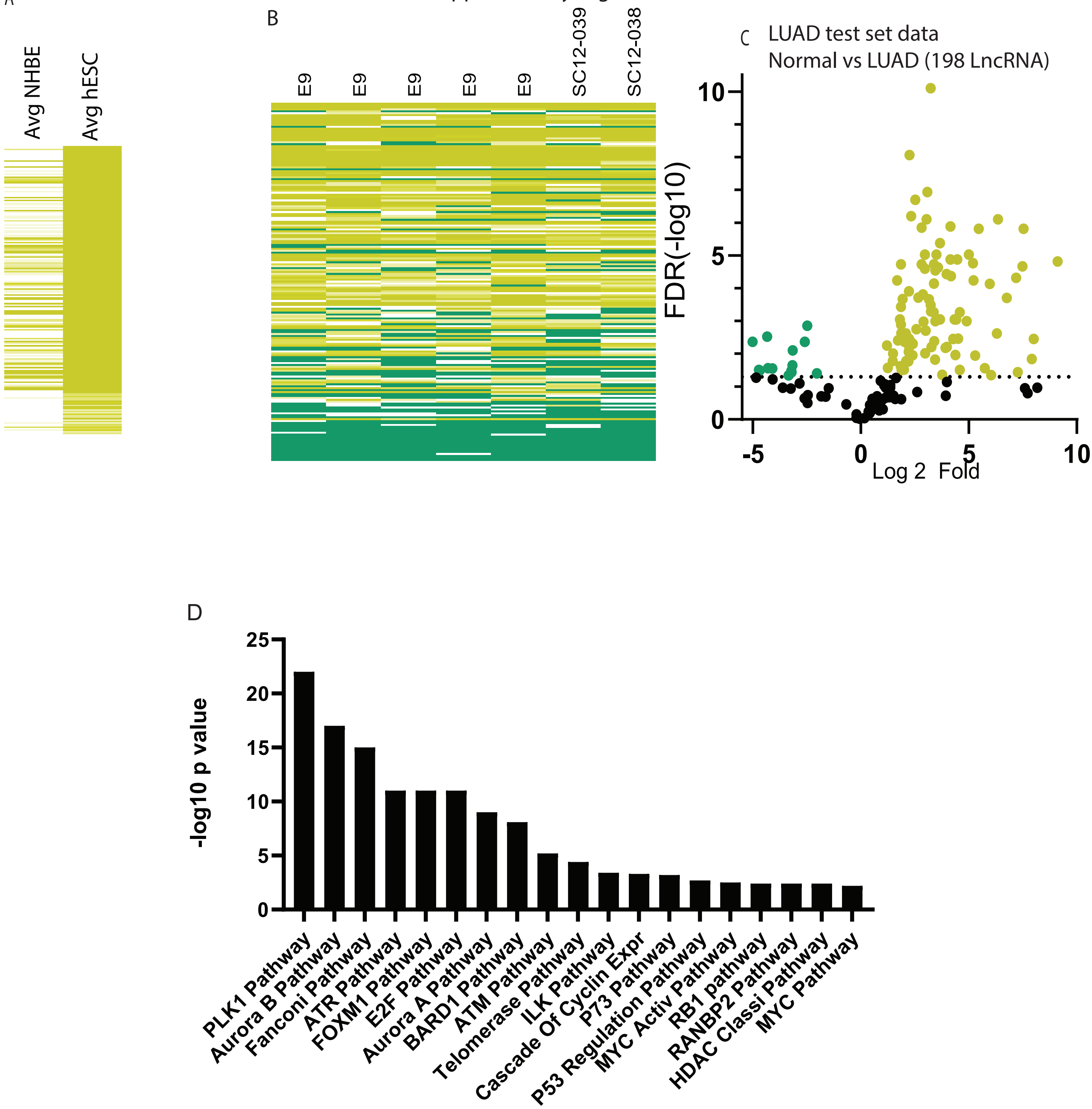

Supplementary Figure 2

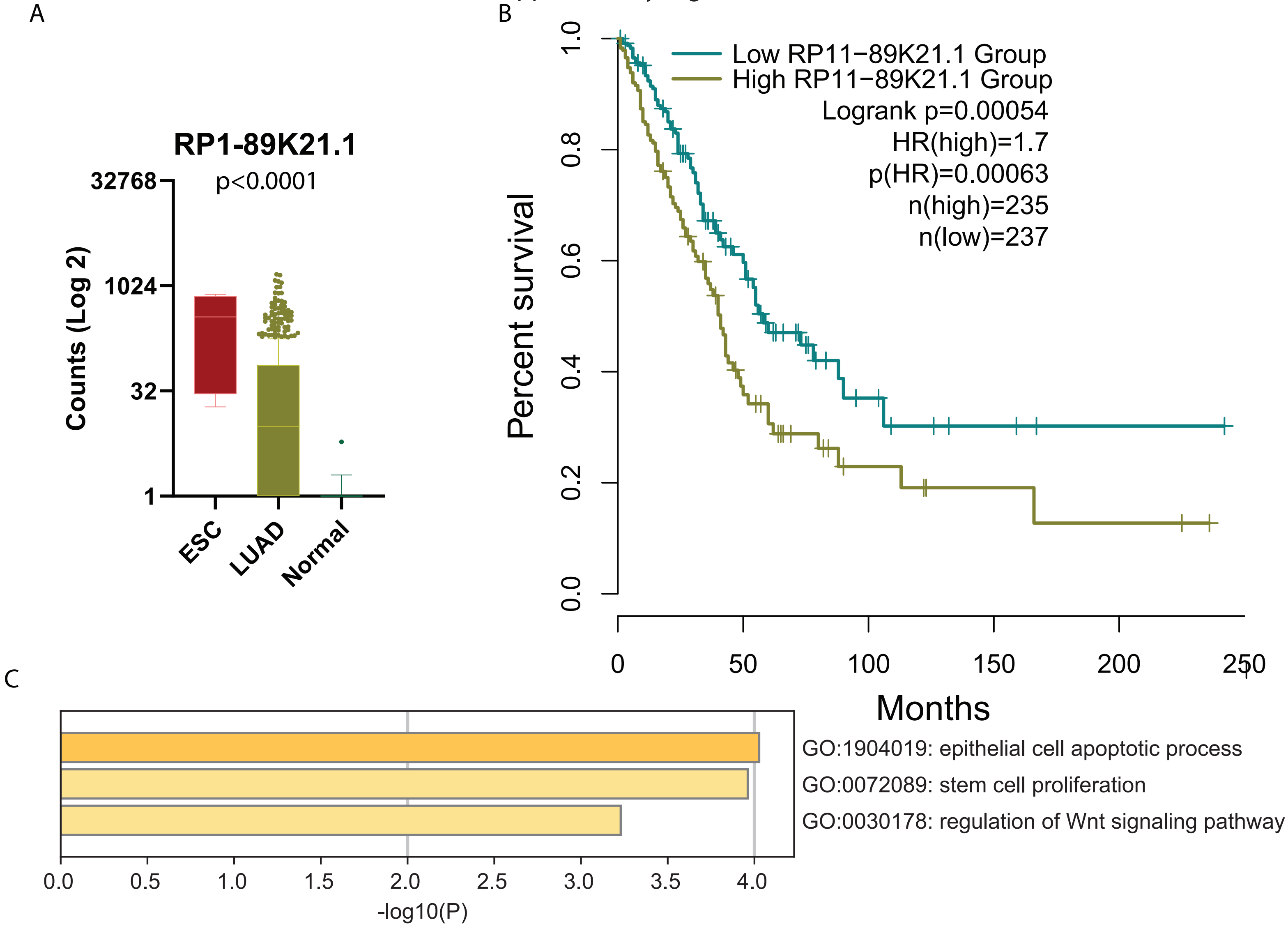

Supplementary Figure 3

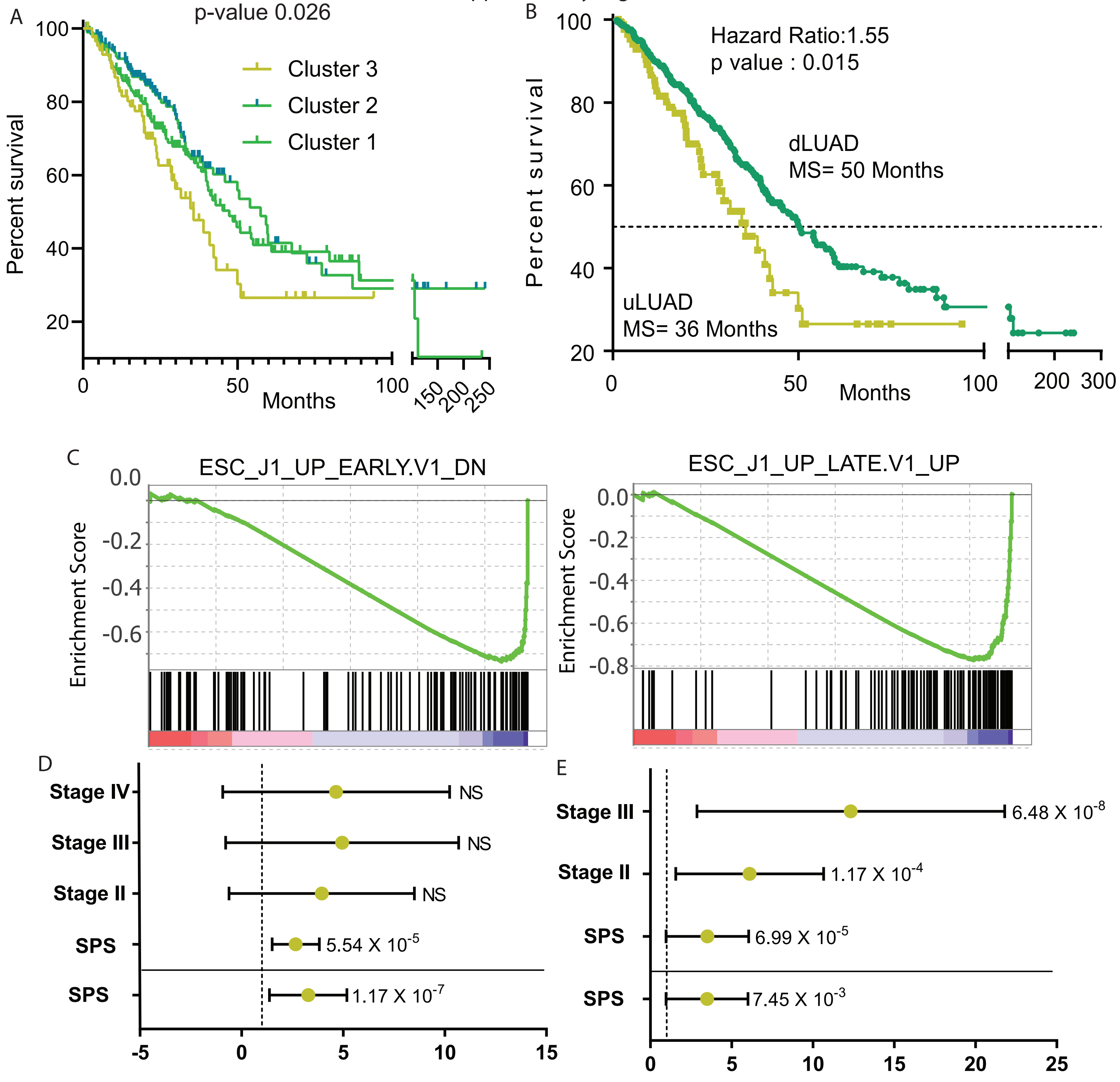

Supplementary Figure 4

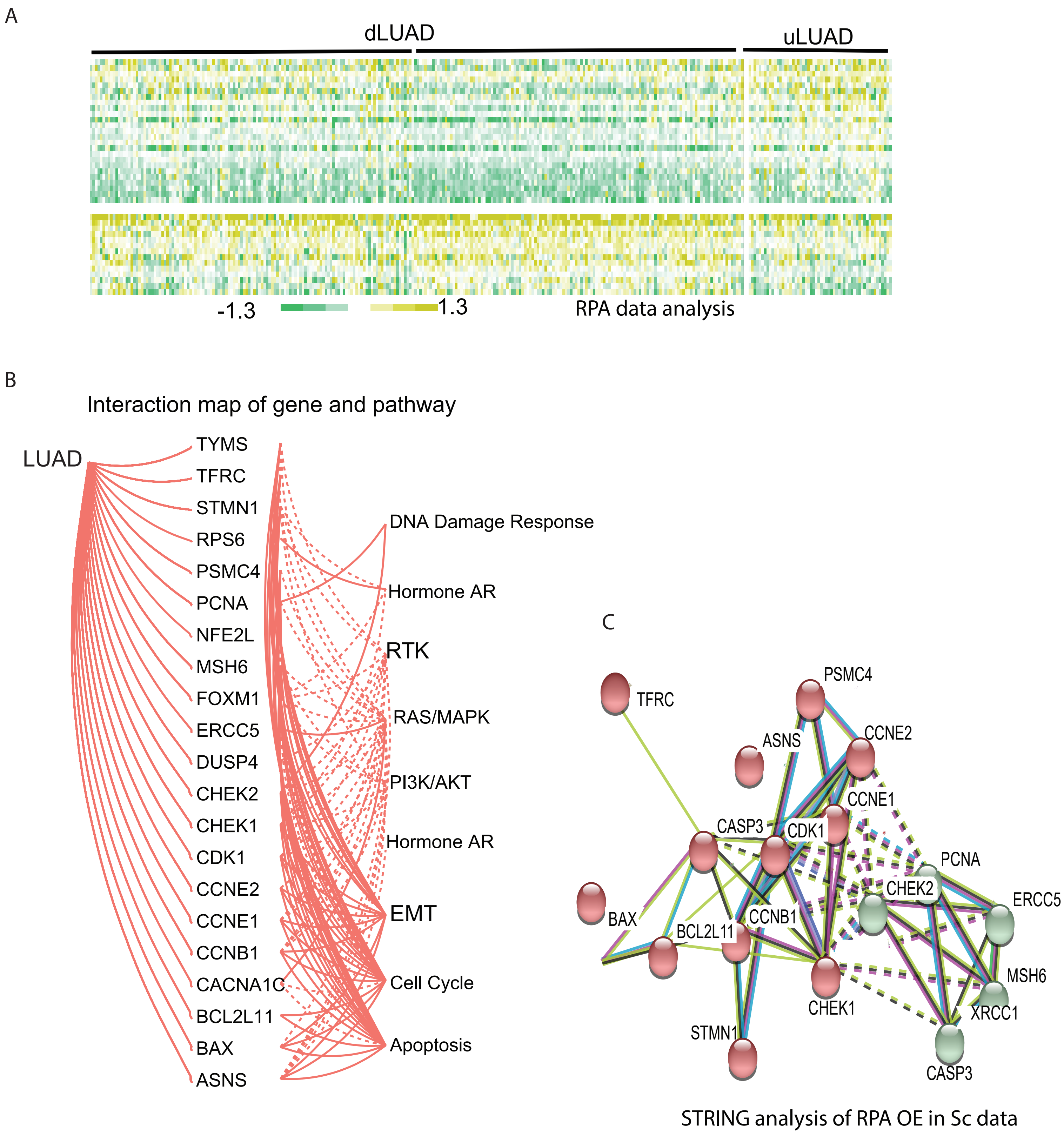

Supplementary Figure 5

A

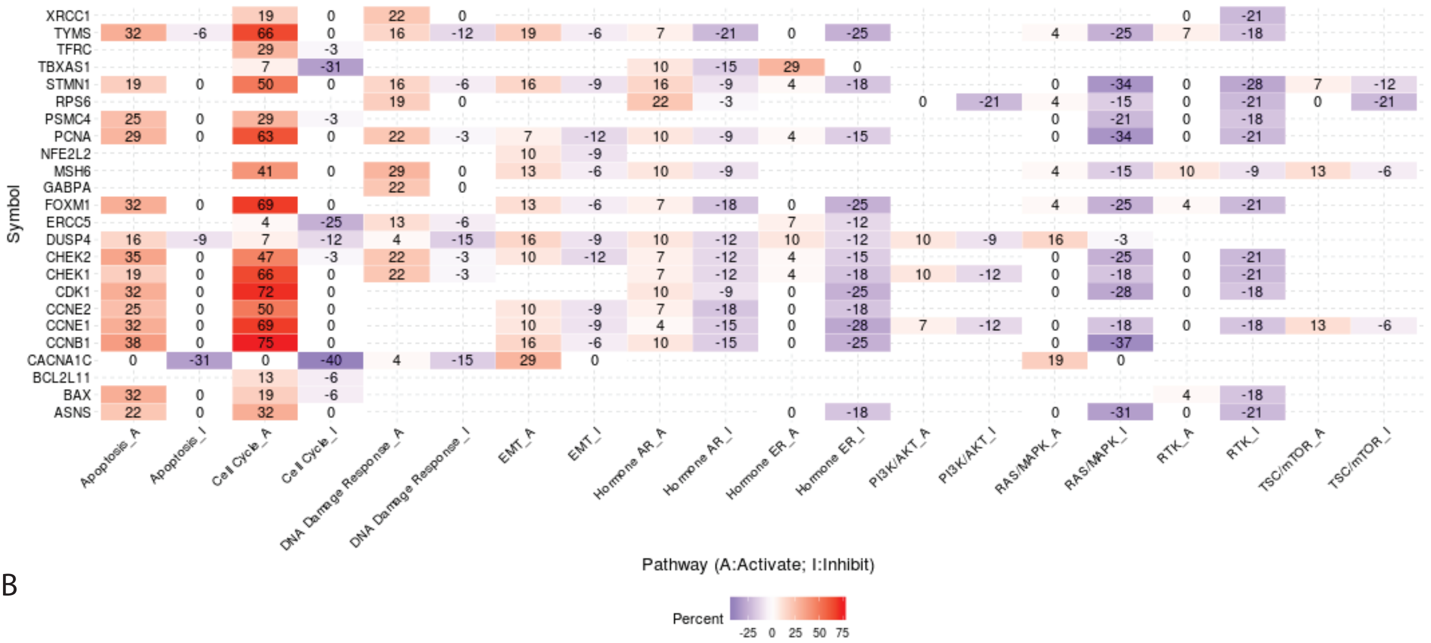

B

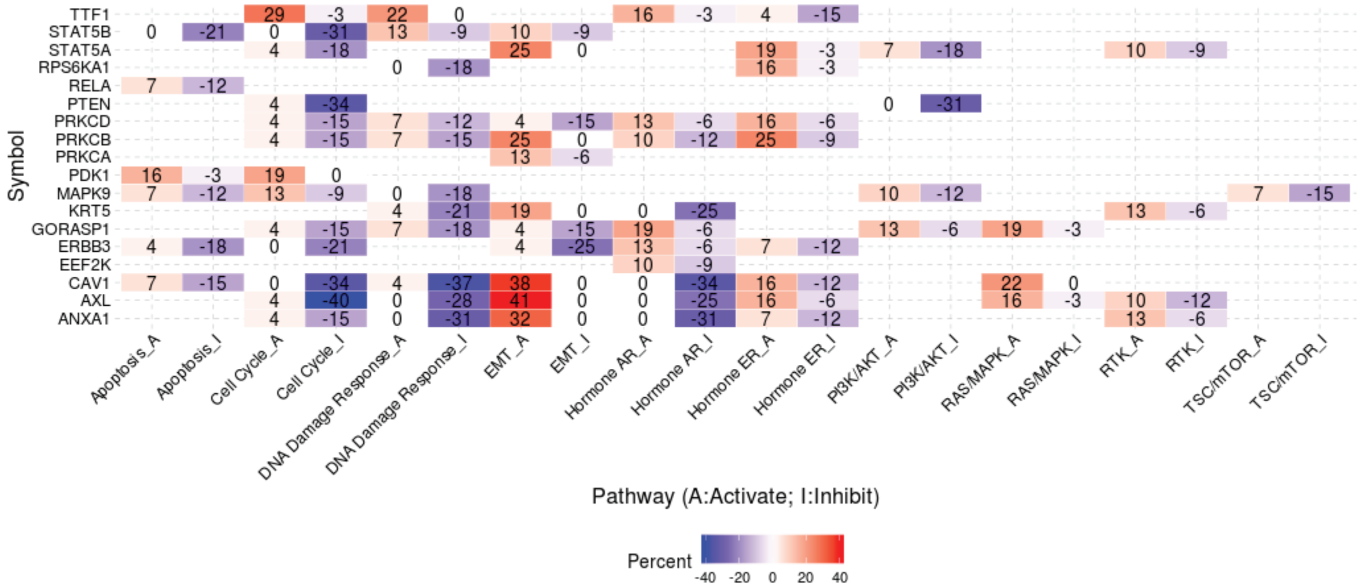

C

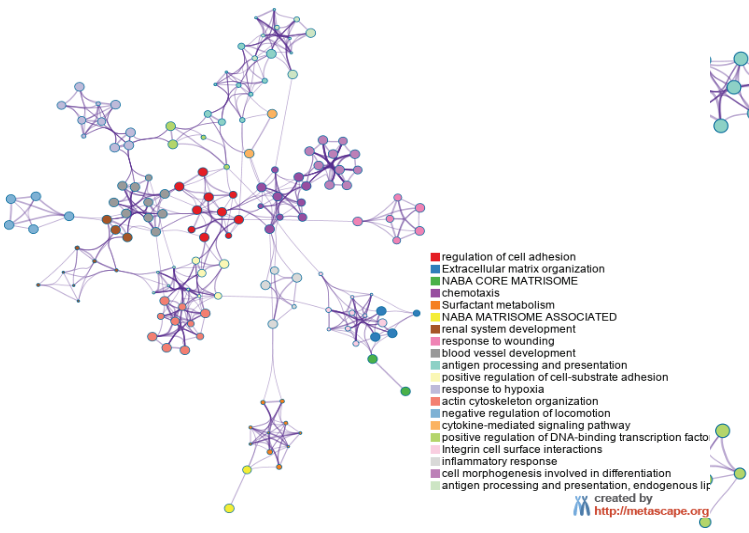

D

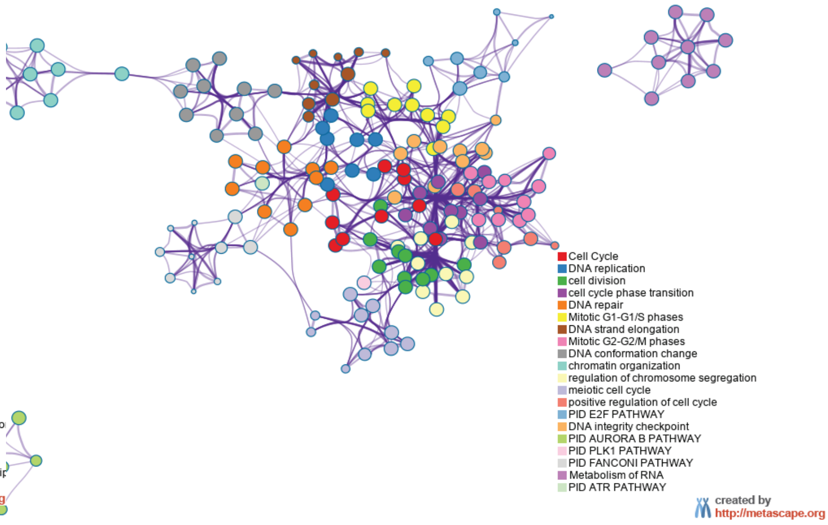

Supplementary Figure 6

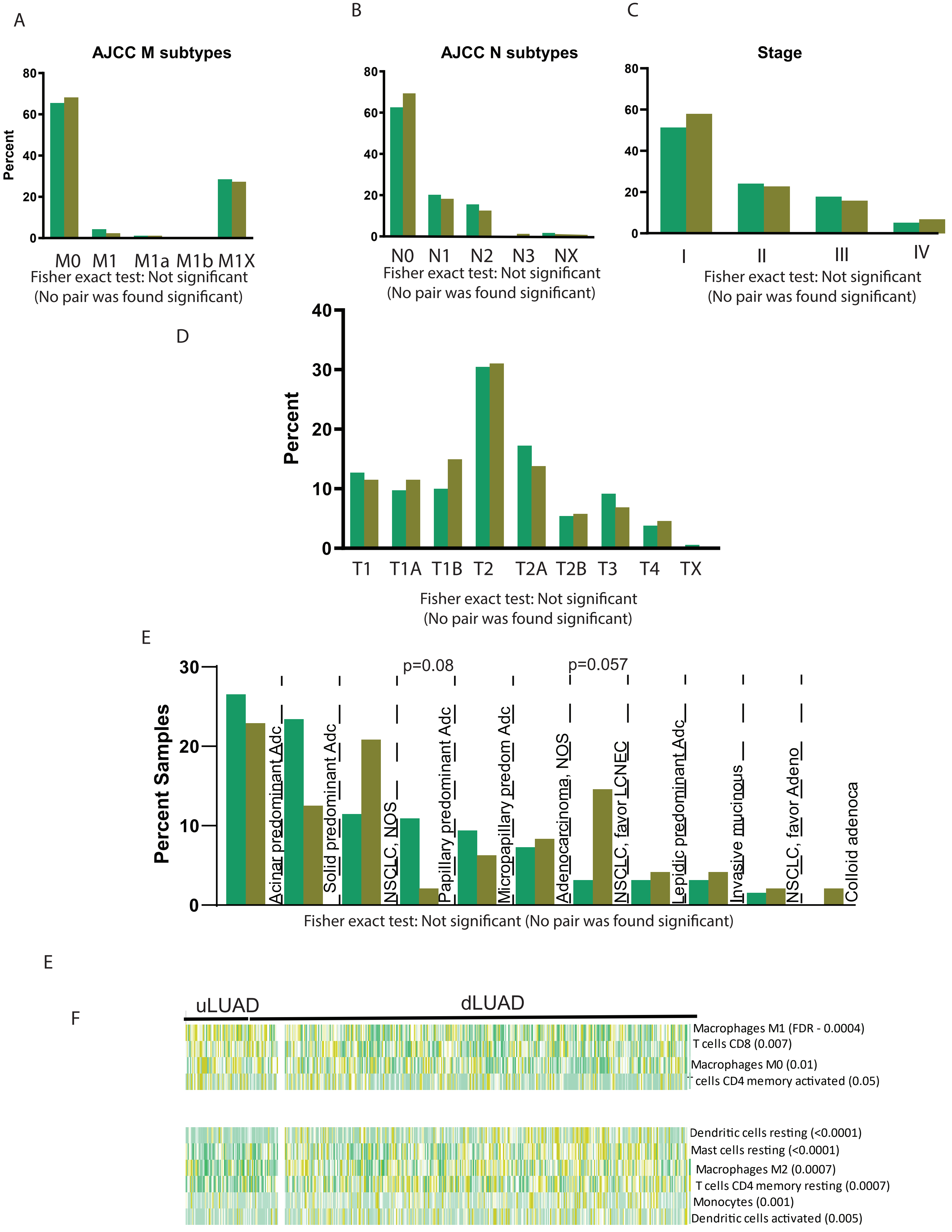

Supplementary Figure 7

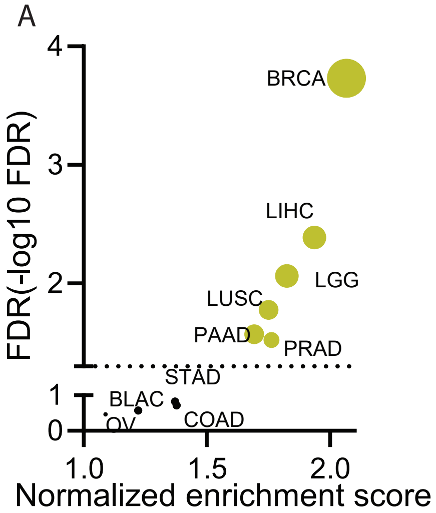

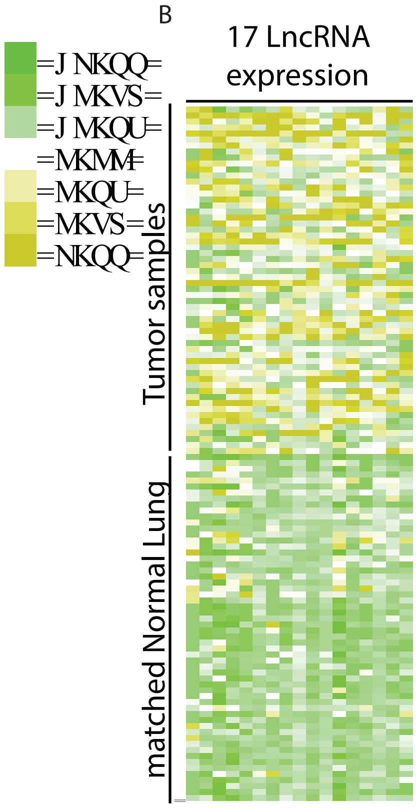

Gene Name	t- test p-value	AUC	ROC P-value
U47924.29	0.0001	0.7	0.0003
RP11-127I20.5	<0.0001	0.83	<0.0001
RP11-55K13.1	<0.0001	0.74	<0.0001
ZNF252P-AS1	<0.0001	0.78	<0.0001
RP11-146F11.5	<0.0001	0.82	<0.0001
RP11-108L7.15	<0.0001	0.9	<0.0001
RP11-485G7.6	<0.0001	0.79	<0.0001
RP11-126L15.4	<0.0001	0.88	<0.0001
RP11-560J1.2	<0.0001	0.82	<0.0001
LINC01572	<0.0001	0.93	<0.0001
CTD-2033A16.3	<0.0001	0.89	<0.0001
UBE2Q1-AS1	<0.0001	0.77	<0.0001
TFAP2A-AS1	<0.0001	0.89	<0.0001
RP11-932O9.10	<0.0001	0.84	<0.0001
LINC01271	<0.0001	0.88	<0.0001
RP11-118K6.3	<0.0001	0.81	<0.0001
RP11-525G13.2	<0.0001	0.73	<0.0001

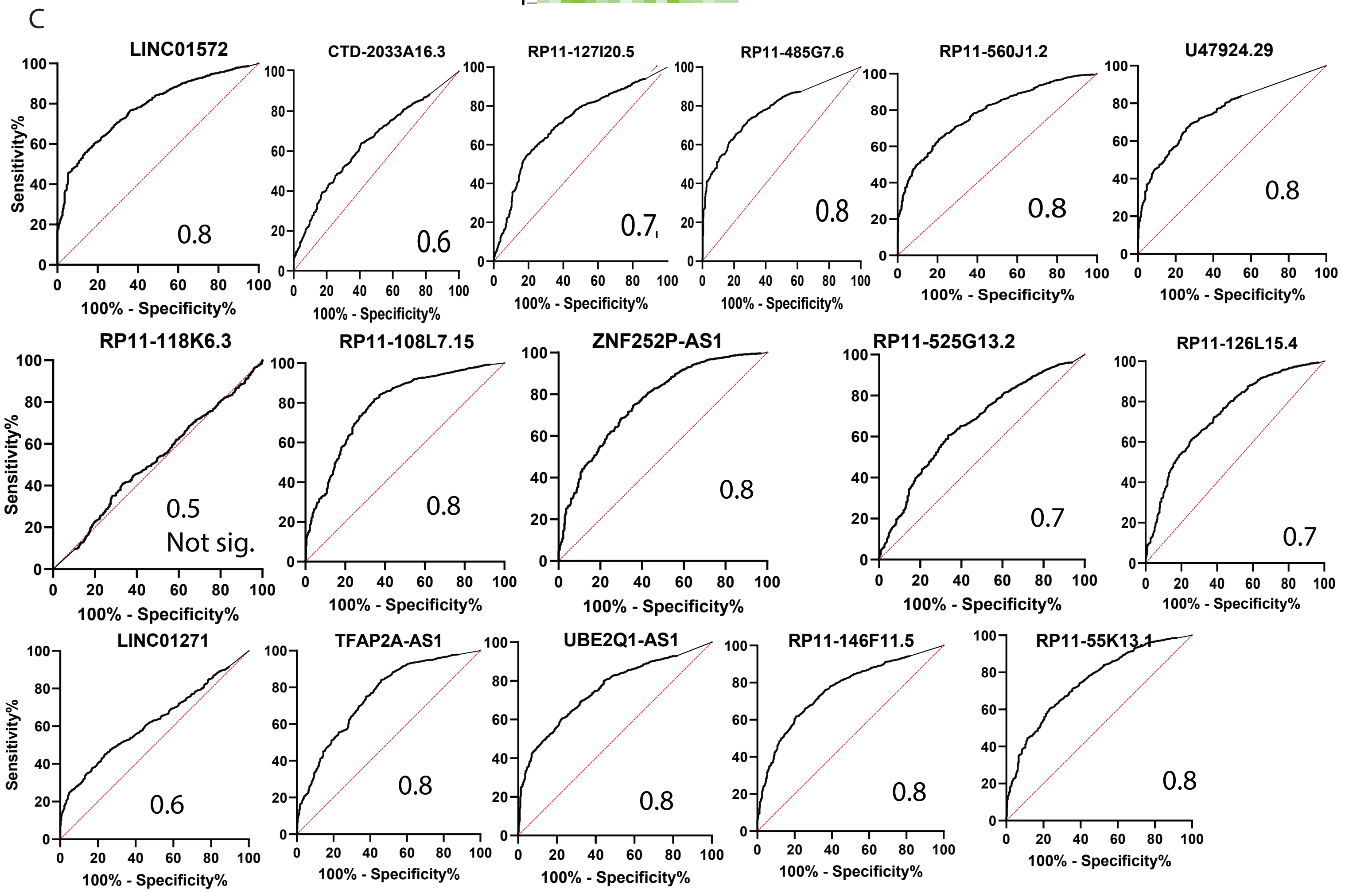

All the pvalues are <0.0001.

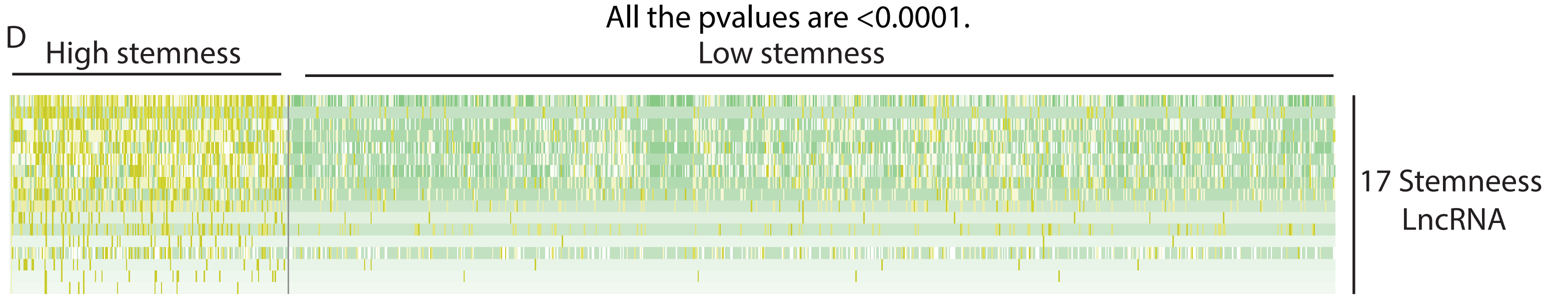

Supplement: Supplementary file 1 [file Data_Sheet_1.PDF]
